# CAR NK92 Cells Targeting BCMA Can Effectively Kill Multiple Myeloma Cells Both In Vitro and In Vivo

**DOI:** 10.3390/biomedicines12010248

**Published:** 2024-01-22

**Authors:** Eunhee Park, Hui-jin Mun, Eunju Seo, Seojin Hwang, Jae Hee Lee, Sukgil Song, Hyeran Sung, Hoi-Yul Kim, Mi-Jin Kwon

**Affiliations:** 1 Department of New Drug Development, Cellgentek Co., Ltd., 110-6, Osongsaengmyeong 2-ro, Heungdeok-gu, Cheongju 28161, Republic of Korea; 2 College of Pharmacy, Chungbuk National University, Cheongju 28160, Republic of Korea

**Keywords:** chimeric antigen receptor, natural killer cell, cancer immunotherapy, multiple myeloma, B-cell maturation antigen (BCMA)

## Abstract

Multiple myeloma (MM) is a hematological malignancy caused by malignant proliferation of plasma cells in bone marrow. Over the last decade, the survival outcome of patients with multiple myeloma (MM) has been substantially improved with the emergence of novel therapeutic agents. However, MM remains an incurable neoplastic plasma cell disorder. In addition, almost all MM patients inevitably relapse due to drug resistance. Chimeric antigen receptor (CAR)-modified NK cells represent a promising immunotherapeutic modality for cancer treatment. In this study, NK92 cells were engineered to express the third generation of BCMA CAR. In vitro, BCMA CAR-engineered NK92 cells displayed higher cytotoxicity and produced more cytokines such as IFN-γ and granzyme B than NK92 cells when they were co-cultured with MM cell lines. Furthermore, BCMA CAR-engineered NK92 cells released significantly higher amounts of cytokines and showed higher cytotoxicity when they were exposed to primary cells isolated from MM patients. The cytotoxicity of BCMA CAR NK92 cells was enhanced after MM cells were treated with bortezomib. Additionally, BCMA CAR NK92 cells exhibited potent antitumor activities in subcutaneous tumor models of MM. These results demonstrate that regional administration of BCMA CAR NK92 cells is a potentially promising strategy for treating MM.

## 1. Introduction

Multiple myeloma (MM) is hematological malignancy characterized by indefinite proliferation of plasma cells in bone marrow. It accounts for 1% of all cancers and approximately 13% of all hematologic malignancies [1,2]. MM patients are commonly treated with proteosome inhibitors such as bortezomib, immunomodulatory agents such as lenalidomide, and monoclonal antibodies such as daratumumab [3]. Among these, bortezomib is approved by the FDA for multiple myeloma. Bortezomib therapy had a significant impact on clinical activity in the treatment of relapsed/refractory multiple myeloma. [4]. Bortezomib reversibly inhibits chymotrypsin-like activity at the β5-subunit and, to a lesser extent, inhibits trypsin-like activity at the β1-subunit. It can also inhibit the post-glutamyl peptide-hydrolyzing activities of the 26S proteasome. Since its introduction, bortezomib has improved the survival and quality of life for patients with newly diagnosed MM. However, most patients eventually relapse [5]. Therefore, more effective therapies are highly needed.

Immunotherapy has recently revolutionized cancer treatment. It constitutes the fourth cornerstone of cancer therapy after surgery, radiation, and chemotherapy. Current immunotherapy explores and harnesses every aspect of the immune system. Immunotherapy using immune checkpoint inhibitors (ICIs) and adoptive cell therapy (ACT) using chimeric antigen receptor (CAR) immune cells are among the most successful immunotherapies [6]. As a result, chimeric antigen receptor T (CAR-T) cells have shown clinical efficacy against numerous hematological malignancies. So far, five CAR T cells have been approved by the FDA [7]. However, many factors contribute to the failure of CAR T-cell therapy, and CAR NK-cell therapy is emerging.

One major advantage of CAR NK cells over CAR T cells is the source of immune cells. CAR NK therapy utilizes “off-the-shelf” ready-to-use CAR NK cells that can be manufactured through mass production and infused to patients at any time. The second major advantage of using CAR NK cells over CAR T cells is that activation of CAR T cells can lead to massive release of inflammatory cytokines which can cause cytokine release syndrome (CRS) and neurotoxicity, whereas the activation of CAR NK cells releases inflammatory cytokines but does not cause CRS [5].

Natural killer (NK) cells play a key role in immune responses to tumors by producing immunoregulatory cytokines and chemokines [8,9]. Recently, chemokines, a family of low-molecular-weight proteins that can act as potent mediators of inflammation, have attracted much interest as they are considered as critical factors [10].

The NK92 cell line is an interleukin-2 (IL-2)-dependent immortalized cell line that has the characteristics of highly active blood natural killer (NK) cells. It is being used in immunology research related to cancer. [11]. Additionally, the safety and efficacy of NK92 cells have been established in clinical trials through clinical responses observed in some cancer patients treated with NK92 [12]. CAR-engineered NK92 cell therapy has various theoretical advantages, including it being an ”Off the shelf” product and immediate availability and lower treatment cost.

In recent years, immune-based therapies such as chimeric antigen receptor (CAR)-engineered immune cells have emerged as a new treatment for MM. One potential target in CAR cell therapy for treatment of MM is B-cell maturation antigen (BCMA) [13]. BCMA is known to play an important role in regulating the survival, proliferation, differentiation, and maturity of B cells in plasma cells. BCMA is a marker for the identification of hematologic malignancies, including multiple myeloma (MM), non-Hodgkin lymphoma (NHL), Hodgkin lymphoma, chronic lymphocytic leukemia, and acute B-lymphoblastic leukemia [14].

So, in this study, we generated CAR NK92 cells armed with an anti-BCMA scFv with high antigen specificity and affinity, which was able to target BCMA. We then investigated the potential of this targeted therapy for the treatment of MM.

## 2. Materials and Methods

### 2.1. Generation and Production of BCMA CAR Lentiviral Construct

An anti-BCMA scFv was derived from DNA sequences encoding a specific monoclonal antibody against BCMA. The anti-BCMA sequence was provided by ABLbio lnc., after signing a material transfer agreement (MTA). The entire anti-BCMA scFv-CD28-4 1BB-CD3ζ fragment was then ligated into a lentiviral vector designated as pCDH-EF1-turboGFP to generate a pCDH-BCMA CAR-turboGFP construct. The lentiviral particles were produced by a commercial vendor (Lugen SCI, Inc., Bucheon, Republic of Korea).

### 2.2. Lentiviral Transduction of NK92 Cells

For lentivirus infection, NK92 cells were adjusted to 1 × 10^5^ cells/mL using α-MEM with IL-2 and 2-mercaptoethanol. They were then loaded into 12-well plates followed by the addition of lentivirus at an MOI of 10 in the presence of Lenti-TD-MAX (Lugen SCI, Inc., Bucheon, Republic of Korea). Cells were then incubated for 24 h. Following this step, the infection protocol was repeated. Starting from day 1 after the second infection, transduced NK92 cells were purified using a FACS Aria II cell sorter (BD Biosciences, San Jose, CA, USA) based on expression of a GFP marker on the cell surface encoded by the vector.

### 2.3. Cell Culture

NK92 cells, part of a human NK-cell line, were purchased from American Type Culture Collection (ATCC, Manassas, VA, USA) and seeded into a T75 flask (1 × 10^5^ cells/mL) using Minimum Essential Medium (MEM) α (Welgene, Gyeongsan, Republic of Korea) supplemented with 12.5% fetal bovine serum (FBS) (Gibco, Waltham, MA, USA), 12.5% horse serum (Gibco), 1% penicillin–streptomycin (Gibco), 1 × 2-mercaptoethanol (Gibco), and 100U/mL Proleukin IL-2 (Bayer HealthCare Pharmaceuticals, Emeryville, CA, USA). The human B-lymphocyte cell lines H929, MM.1R, U266, and RPMI8226 and lymphoblast cell line K562 were purchased from American Type Culture Collection (ATCC, Manassas, VA, USA). H929, MM.1R, RPMI8226, and K562 cells were cultured using RPMI 1640 (Welgene, Republic of Korea) supplemented with 10% fetal bovine serum and 1% penicillin–streptomycin. U266 cells were cultured using RPMI 1640 supplemented with 15% fetal bovine serum and 1% penicillin–streptomycin. All cells were incubated at 37 °C in a humidified atmosphere with 5% CO_2_.

### 2.4. Flow Cytometry Analysis of Cell Phenotype

Anti-NKG2D-APC (#130-117-718) and anti-DNAM-1-APC (#130-124-241) were purchased from Miltenyi Biotec (Auburn, CA, USA) and anti-CD16-Pacific Blue (#562874) was purchased from BD Bioscience (San Jose, CA, USA). Cells were stained with antibodies for 20 min at 4 °C. After 20 min of staining, unincorporated dye was removed by washing with 1% FBS containing PBS. Samples were then centrifuged at 1000 rpm for 3 min and the pellets were resuspended in 500 μL of 1% FBS containing PBS. Measurements were performed on a Novocyte flow cytometer (Novocyte, Agilent Technologies, Santa Clara, CA, USA).

### 2.5. Western Blot

Cells were harvested using a Passive Lysis Buffer (#E1941, Promega, Madison, WI, USA). Protein concentrations of cell lysates were determined using a Pierce BCA protein assay kit (#23225, Thermo Fisher Scientific, Rockford, IL, USA). Samples were resolved with 12% sodium dodecyl sulfate–PAGE gels and transferred to polyvinylidene difluoride (PVDF) membranes (Bio-Rad, Hercules, CA, USA). Membranes were blocked with a 5% nonfat milk solution in TBST buffer (20 mM Tris–HCl, pH 7.4, 500 mM NaCl, 0.1% Tween 20) for 2 h at room temperature. Blots were then incubated with CD3ζ antibodies (diluted 1:1000) (phosphor Y83, Abcam, Boston, MA, USA) in TBST overnight at room temperature. Equal loading was confirmed with an anti-β-actin (Sigma-Aldrich Co. LLC., St Louis, MO, USA). After blots were washed three times in TBST, they were incubated with an anti-rabbit secondary antibody in TBST for 1 h at room temperature. Finally, immunoblots were detected by SuperSignal^®^ West Pico Chemiluminescent Substrate (#34580, Thermo Fisher Scientific, Waltham, MA, USA) and exposure to an image analysis system (Amersham Imager 600, GE Healthcare, Uppsala, Sweden).

### 2.6. Cytotoxicity

Target cells (K562, H929, MM.1R, U266, RPMI8226) were stained with a 50 nM CellTrace™ CFSE dye (#21888, Sigma-Aldrich, St Louis, MO, USA) at 37 °C for 15 min. Target cells were plated at a density of 2 × 10^4^ cells/well in a 96-well U-bottom plate and co-cultured with effector cells (Con NK92, CAR NK92) at an E/T ratio of 1:1 for 4 h at 37 °C. After 4 h of reaction, plates were centrifuged and washed using PBS containing 1% FBS. Target cell cytotoxicity against effector cells were assessed using 7-amino-actinomycin (7-AAD) (#559925, BD Biosciences, San Jose, CA, USA). 7-AAD-positive cells were analyzed using flow cytometry (Novocyte, Agilent Technologies, Santa Clara, CA, USA). Cytotoxicity was calculated using the following equation: Co-culture 7-AAD positive (%) − Target cell 7-AAD positive (%) − effector cell 7-AAD positive (%).

### 2.7. Enzyme-Linked Immunosorbent Assay (ELISA)

Target cells (K562, H929, MM.1R, U266, RPMI8226) were plated into a 96-well U-bottom plate at a density of 2 × 10^4^ cells/well and co-cultured with effector cells (Coc NK92, CAR NK92) at a ratio of 1:1 for 4 h at 37 °C. After 4 h of co-culturing, plates were centrifuged at 1000× *g* for 5 min and supernatants were harvested for ELISA. The concentration of IFN-γ in each well was determined using a human IFN-γ ELISA kit (#421203, Biolegend, San Diego, CA, USA). The concentration of granzyme B was determined using a human granzyme B ELISA kit (#DY2906, R&D system, Minneapolis, MN, USA). All groups were analyzed in duplicate.

### 2.8. Live Cell Imaging (LCI)

Target cells were stained with 5 µM CellTracker™ Green CMFDA dye (#C7025, Thermo Fisher Scientific) for 30 min at 37 °C, washed three times, and maintained with complete medium. Target cells were seeded into a 96-well plate at a density of 2 × 10^4^ cells/well. They were then added to effector cells immediately before the start of imaging at a 1:1 E:T ratio (2 × 10^4^ cells/well). LCI was performed at 37 °C with 5% CO_2_ in a total volume of 300 µL complete medium without IL-2, containing propidium iodide (Thermo Fisher Scientific) for dead cells. Time-lapse imaging was performed using an ImageXpress Nano Automated Imaging System (Molecular devices, San Jose, CA, USA). Images were acquired every 5 min for 4 h in a single z plane for transmitted light and fluorescent channels.

### 2.9. CD138-Positive Cell Isolation from Bone Marrow of MM Patients

Bone marrow specimens were obtained from multiple myeloma patients at the Department of Hematology–Oncology, Chungbuk National University Hospital. This study was approved by the Medical Ethics Committee of the Chungbuk National University Hospital (IRB No. 2020-01-002-001). Human mononuclear cells were isolated from bone marrow by density gradient centrifugation (25 min, 500× *g*) using Ficoll–Paque medium (d = 1.078, # 17-1440-03, Cytiva, Marlborough, MA, USA) and washed twice with phosphate-buffered saline (Gibco, Waltham, MA, USA). CD138-positive cells from peripheral blood were isolated using CD138 microbeads (#130-105-961, Miltenyi Biotec, Auburn, CA, USA) according to the manufacturer’s instructions. Briefly, PBMC at a concentration of 1 × 10^8^ cells/mL were incubated with CD138 microbeads at a titer of 1:5. After 30 min of incubation at 4 °C, cells were washed once with PBS containing 5 mm EDTA and 0.5% BSA (PBS/EDTA/BSA). After resuspending cells in 2 mL PBS/EDTA/BSA, cells were separated using two sequential MS columns (Miltenyi Biotec).

### 2.10. In Vivo Efficacy Studies

All protocols for animal studies were reviewed and approved by the Osong Medical Innovation Foundation Laboratory Animal Center, KBIO (Permit NO. KBIO-IACUC-2019-112). Six-week-old female NOD mice were purchased from KOATECH Co., Ltd. (Pyeongtaek, Republic of Korea). The housing conditions of the mice included a constant temperature of 23 °C with ventilation of 10–20 times per hour. Every day, 12 h of illumination and 12 h of darkness were maintained. Food and water were sterilized by high-pressure steam. Each mouse was inoculated in the right flank subcutaneously with 3 × 10^6^ NCI-H929^luc+^ cells (NCI-H929 cells expressing luciferase) on day 0. After tumor cell injection, when tumor size reached 100 mm^3^, mice were randomly assigned into three groups, (1) an untreated group, (2) a Con NK92 group, and (3) a CAR NK92 group. At 24 h after tumor cell injection, mice were intravenously injected with 1.5 × 10^7^ Con NK92 cells or CAR NK92 cells in 300 µL PBS or the same volume of PBS without cells. The NK92 cell treatment was repeated every three days until the experiment was terminated. Before NK92 injection, NK92 and CAR NK92 cells were irradiated with 10 Gy (X-RAD IR160, PRECISION, Madison, CT, USA). During the treatment, the tumor size and body weight of mice were monitored every three days. Tumors were assessed with a caliper at their greatest length and width to estimate tumor volume. Tumor volume was calculated using the following formula: tumor volume = (length × width)^2^/2. On day 18, the experiment was terminated. After mice were sacrificed, tumors were separated and their weights were measured.

### 2.11. Statistical Analysis

All results were confirmed with at least three independent experiments. Data from one representative experiment are shown. All data were analyzed using GraphPad Prism 5.0 software (GraphPad Inc., San Diego, CA, USA). Quantitative data are shown as means ± standard deviation. Significance of statistical analysis was determined with two-tailed, unpaired Student’s *t*-tests. *p*-values less than 0.05 were considered significant.

## 3. Results

### 3.1. Generation of BCMA CAR NK92 Cells

**Figure 1:**
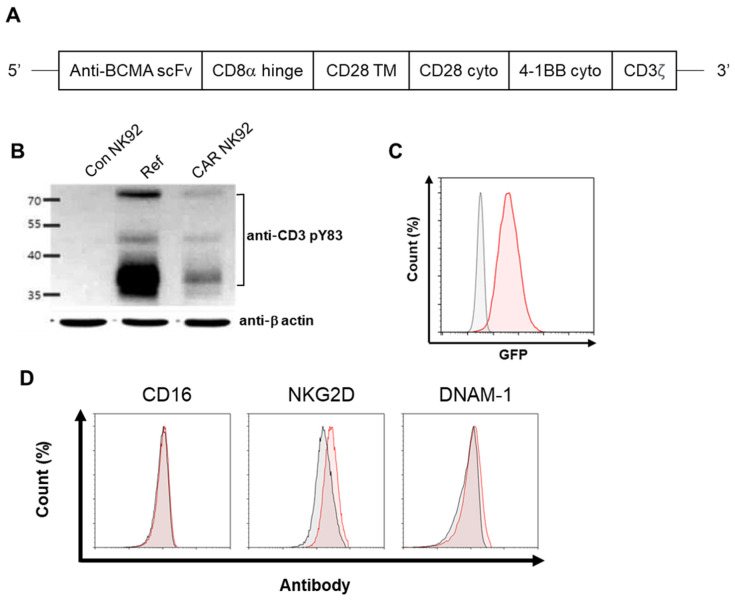
Generation of BCMA CAR NK92 cells. (**A**) Schematic representation of CAR molecules. (**B**) CAR expression in transduced BCMA CAR NK92 cells was analyzed by Western blot analysis. (**C**) Percentages of BCMA CAR NK92 cells were detected by flow cytometry. GFP served as a marker of CAR expression. (**D**) NK-cell-activating receptors (NKG2D, CD16, DNAM1) in NK92 and CAR NK92 cells were measured by flow cytometry.

We generated a third-generation BCMA CAR construct in the pCDH lentiviral backbone. This construct sequentially contained BCMA scFv, CD8α hinge, CD28, 4-1BB, and CD3ζ (Figure 1A). NK92 cells were transduced with CAR-expressing lentiviruses and sorted based on expression of GFP by the vector. Western blot analysis was performed using anti-CD3 to recognize the CD3ζ portion. As shown in Figure 1B, BCMA CAR was expressed on the surface BCMA CAR-transduced NK92 cells. However, its expression was undetectable on NK92 cells. Among the lanes of the blot, the ref was an antibody identical to the scFv of the CAR construct we produced. Additionally, we confirmed GFP using flow cytometry after sorting (Figure 1C). NK cells express several activating and inhibitory receptors that can recognize altered expression of proteins on target cells and control the cytolytic function [15]. NKG2D, NCRs, DNAM1, and CD16 are the best characterized activating NK cell receptors implicated in immune responses against cancer [16,17]. Next, we explored whether BCMA CAR expression could confer NK92 cells with changed NK-cell-activating receptors. No significant phenotypic differences were observed between NK92 and BCMA CAR NK92 cells in the flow cytometry performed (Figure 1D). These data showed that BCMA CAR expression allows specific BCMA-targeting activity while maintaining native NK-expressed receptor function.

### 3.2. BCMA CAR NK92 Cells Exhibit Cytotoxicity against MM Cell Lines

**Figure 2:**
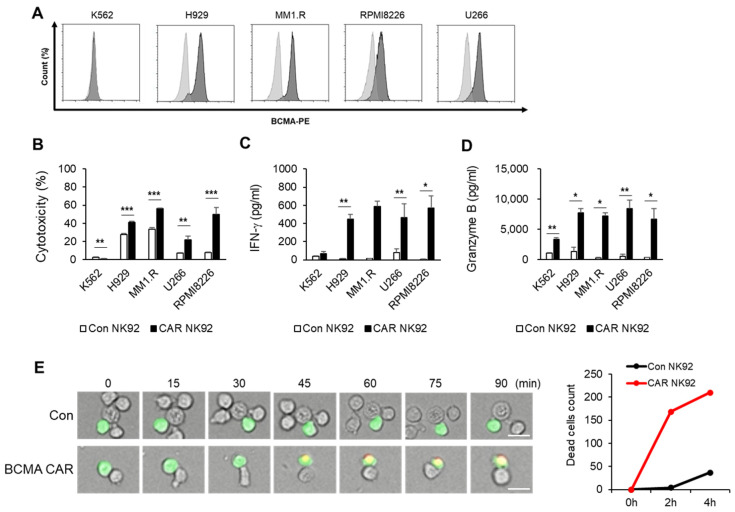
BCMA CAR NK92 cells are cytotoxic to MM cell lines. (**A**) Surface BCMA expression in human MM cell lines was assessed by flow cytometry. MM cell lines were immunostained with anti-BCMA (dark gray) or isotype control human IgG (light gray). (**B**) Cytotoxicities of NK92 and CAR NK92 cells against MM cell lines were measured by flow cytometry after a 4 h co-incubation with MM target cells at an E/T ratio of 1:1. Released (**C**) IFNγ and (**D**) granzyme B were analyzed using ELISA kits after a 4 h co-incubation with MM target cells at an E/T ratio of 1:1. (**E**) H929 cells were co-incubated with NK92 and CAR NK92 for 4 h. H929 cells were stained with CellTracker™ Green CMFDA Dye and PI. Live (green) and dead (red) cells were detected by live/dead assay and visualized by confocal microscopy. scales bars: 20 μm. *, *p* ≤ 0.05; **, *p* ≤ 0.01; ***, *p* ≤ 0.001.

To assess the surface expression of BCMA in MM cell lines, cells were stained with a BCMA-specific antibody, followed by flow cytometric analysis. MM cell lines exhibited substantial BCMA surface expression, while K562, the lymphoblast cell line, had no such expression (Figure 2A). To evaluate the cytotoxic potential of BCMA CAR NK92 cells against MM cell lines, cytotoxicity was assayed by flow cytometry using CFSE/7AAD staining. BCMA CAR NK92 cells showed higher cytotoxicity to MM cells than control NK92 cells when they were co-cultured with MM cell lines. However, they showed no cytotoxicity against the K562 lymphoblast cell line (Figure 2B). Activated NK cells can secrete a variety of cytokines such as IFN-γ, TNF-α, GM-CSF, and granzyme B. Cytokines secreted by NK cells plays a crucial role in antiviral, antibacterial, and antitumor activities [15,18]. Thus, we explored whether BCMA CAR NK92 cells might enhance cytokine production. A cytokine-secreting assay revealed that IFN-γ (Figure 2C) and granzyme B (Figure 2D) levels were significantly increased in supernatants after BCMA CAR NK92 cells were co-cultured with MM cell lines for 4 h. However, they were not changed in supernatants after control NK92 cells were co-cultured with MM cells lines. We also analyzed NK–MM cell line interactions in real time through live cell imaging. H929 cells were fluorescently stained with a CellTracker™ Green CMFDA Dye for discriminating NK cells. After cells were stained with PI reagent for dead cell discrimination, cell images were captured every 5 min over 4 h using a confocal microscope. As expected, BCMA CAR NK92 cells rapidly killed H929 cells whereas NK92 cells induced death of H929 cells slowly after 2 h of co-culture (Figure 2E). These results demonstrate that generated CAR NK cells specifically react to BCMA with increased cytotoxicity compared to control NK92 cells.

### 3.3. BCMA CAR NK92 Cells Active against Primary MM Cells

**Figure 3:**
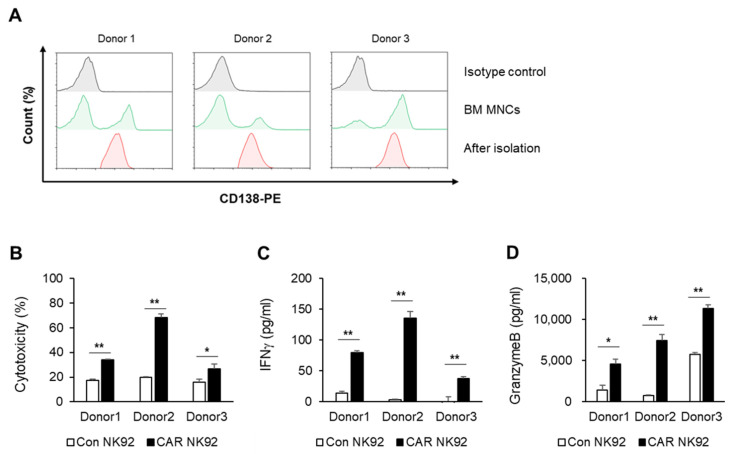
BCMA CAR NK92 cells active against primary MM cells. (**A**) CD138^+^ cells isolated from BM MNCs of three patients were available for flow cytometry. Histogram shows expression of CD138 (green) in BM MNCs compared with isotype control (gray). After CD138^+^ cells were isolated using microbeads, histogram showed expression of CD138 (red). (**B**) Cytotoxicities of NK92 and CAR NK92 cells against CD138^+^ cells obtained from MM patients were measured by flow cytometry after a 4 h co-incubation with target cells at an E/T ratio of 1:1. Released amounts of (**C**) IFNγ and (**D**) granzyme B were analyzed using ELISA kits after a 4 h co-incubation with target cells at an E/T ratio of 1:1. *, *p* ≤ 0.05; **, *p* ≤ 0.01.

CD138 expression is a hallmark of plasma cells and MM cells [19]. To further validate our results, CD138-positive cells obtained from three patients with MM were used. CD138-positive cells were isolated from bone marrow samples of MM patients using microbeads. Although the number of CD138-positive cells varied depending on patients, it was found that CD138-positive cells were completely isolated after separation using microbeads (Figure 3A). To determine whether cytotoxicity of BCMA CAR NK92 cells was increased compared to control NK92 cells, CD138-positive cells were co-cultured with NK92 or BCMA CAR NK92 cells. The cytotoxicity of BCMA CAR NK92 was increased compared to that of control NK92 cells after they were co-cultured with primary MM cells (Figure 3B). BCMA CAR NK92 cells also released significantly higher amounts of IFN-γ (Figure 3C) and granzyme B (Figure 3D) than NK92 cells when they were co-cultured with CD138-positive cells. These data strongly corroborated previous results on MM cell lines and further confirmed the potential of CAR NK92-TRAIL cells for treating multiple myeloma patients.

### 3.4. Bortezomib Increases Sensitivity of MM Cells to BCMA CAR NK92-Mediated Killing

**Figure 4:**
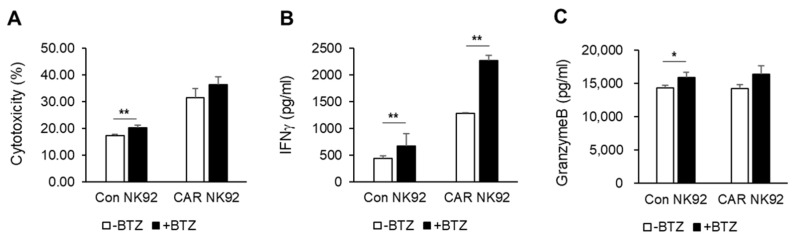
Bortezomib increases sensitivity of MM cells to BCMA CAR NK92-mediated killing. U266 cells were treated with vehicle or bortezomib (10 nM) for 12 h and then co-cultured in the presence of control NK92 or CAR NK92 cells for 4 h. (**A**) Cytotoxicities of NK92 and CAR NK92 cells against U266 cell lines were measured by flow cytometry after a 4 h co-incubation at an E/T ratio of 1:1. Released amounts of (**B**) IFNγ and (**C**) granzyme B were analyzed using ELISA kits after a 4 h co-incubation at an E/T ratio of 1:1. *, *p* ≤ 0.05; **, *p* ≤ 0.01.

In an attempt to increase the efficiency of BCMA CAR NK92 cells, we investigated ways to sensitize MM cells to BCMA CAR NK92 cells. One way was to use a combination therapy with drugs used for MM patients. Bortezomib was the first proteasome inhibitor approved for treating myeloma. It represents a step forward in the management of these patients [20]. We evaluated the ability of BCMA CAR NK92 cells to mediate cytotoxic activity against bortezomib-treated MM cell lines. MM cell lines were treated with bortezomib (10 nM) for 12 h. Cytotoxicities of NK92 cells and BCMA CAR NK92 cells against bortezomib-treated MM cell lines were then measured by flow cytometry after a 4 h co-incubation with MM target cells at an E/T ratio of 1:1. Cytotoxicities of NK92 cells and BCMA CAR NK92 cells against bortezomib-treated MM cell lines were slightly increased compared to than those against untreated MM cell lines (Figure 4A). Moreover, the release of IFN-γ from bortezomib-treated MM cell lines was significantly increased compared to that from untreated MM cell lines (Figure 4B). Control NK92 cells and BCMA CAR NK92 cells released slightly higher amounts of granzyme B when they were incubated with bortezomib-treated MM cell lines than when they were incubated with untreated MM cell lines (Figure 4C). Collectively, these data confirmed the synergistic therapeutic potential of combining anti-BCMA with bortezomib.

### 3.5. BCMA CAR NK92 Cells Induce Tumor Remission in In Vivo MM Models

**Figure 5:**
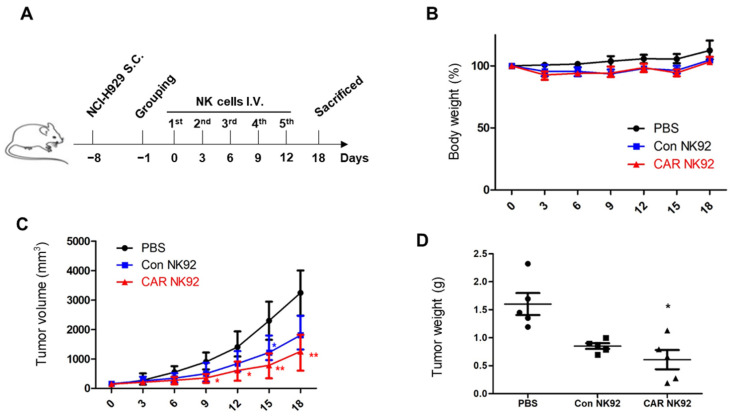
BCMA CAR NK92 cells induce tumor remission in In vivo MM models. (**A**) Experimental outline for the animal study. Three groups of NOD mice (*n* = 5 per group) received s.c. injection of NCI-H929 luc cancer cells followed by i.v. injection of PBS, control NK92 cells, or BCMA CAR NK92 cells as indicated. (**B**) Statistical chart of mouse body weight. (**C**) Tumor volume in mice subcutaneously injected with NCI-H929 was calculated as (length × width)^2^/2. (**D**) Tumor weights of NCI-H929 subcutaneous models at the end point. *, *p* ≤ 0.05; **, *p* ≤ 0.01.

To examine the tumor regression capability of BCMA CAR NK92 cells in vivo, subcutaneous MM models were established by injecting NCI-H929 cells into the right flank of each mouse. The experimental scheme is shown in Figure 5A. A week later, when tumor volumes reached 100 mm^3^, mice were randomized into three groups. Then, 1.5 × 10^7^ control NK92 cells or BCMA CAR NK92 cells were injected intravenously (i.v.) every three days. The experiment ended on the 18th day. Body weights of mice in the two groups injected with NK were slightly decreased compared to those in the group injected with PBS, although such decreases were not clinically significant (Figure 5B). It was discovered that the anti-tumor effect of BCMA CAR NK92 cells was stronger than that of control NK cells. In the BCMA CAR NK92 group, the calculated tumor volume decreased steadily compared with that in the control NK92 group (Figure 5C). Correspondingly, the measured average tumor weight in the BCMA CAR NK92 group was significantly lower than that in the control NK92 group (Figure 5D). These results confirmed that BCMA CAR NK92 cells had a great potential for suppressing the growth of BCMA-positive MM cells in subcutaneous models.

## 4. Discussion

Multiple myeloma (MM) is the second most common hematological malignancy [1]. In a therapeutic environment of MM therapy, B-cell mature antigen (BCMA), one of the most specific and highly expressed antigens in bone marrow cells, occupies promising targets [21]. In CAR T-cell immunotherapy, the CAR strategy targeting BCMA is known to be innovative by combining the target specificity of mAB and the cell toxicity of T cells [22]. Most recent in March 2021, FDA approved the use of Abecma^®^, a BCMA, for treating MM. However, many factors such as cost, cytokine release syndrome, and individuality of patients can contribute to failure of CAR T-cell therapy.

NK cells are a group of cytotoxic lymphocytes of the innate immune system that can mount a rapid response to non-self cells [15]. CAR NK cells can overcome the disadvantages of CAR T cells mentioned above. Additionally, NK92 NK cells can proliferate indefinitely. Thus, they can be developed as stable, easy-to-use “off-the-shelf” cells [12]. In the present study, we constructed a third-generation CAR against BCMA and developed a CAR-modified NK92 cell line. Although NK92 cells require an irradiation step before clinical use due to the immortality of the cell line and resulting safety concerns, they have advantages of being easily scalable and highly available as an “off-the-shelf” product, which can significantly reduce both the treatment cycle and treatment cost. The goal of this study was to generate BCMA CAR NK92 cells to cause MM cell death and determine whether they could be used as a potential therapy. To demonstrate the function of BCMA CAR NK cells, we tested CAR constructs using multiple myeloma cell lines. As shown in Figure 2, targeting BCMA can selectively cause death of myeloma cells that overexpress the BCMA antigen. However, cell death was not observed for cells that did not express BCMA, such as K562 cells. IFN-γ is known to directly enhance NK cell-mediated induction of cancer cell death due to apoptosis and cytolysis. Our results showed that levels of IFN-γ and granzyme B were higher in BCMA CAR NK cells than in control NK92 cells. This phenomenon was confirmed not only in MM cell lines but also in primary MM cells. Experiments using primary MM cells isolated from bone marrow samples of MM patients showed the same results as those using cell lines. BCMA CAR NK cells exhibited significantly increased cytotoxicity to MM cells isolated from BM samples of patients with MM and released more cytokines than control NK cells (Figure 3).

To enhance the efficiency of CAR NK cells against MM, combination therapy with already used drugs can be considered. We used bortezomib, the most commonly used proteasome inhibitor in patients with MM. Bortezomib activates immune responses through upregulating the expression of NK-cell-activating receptors NKG2D, DNAM-1, and TRAIL [11]. In vitro studies have shown that bortezomib and BCMA CAR NK92 cells exhibited synergistic effects by releasing cytokines such as IFN-γ. However, the mechanism involved was not revealed in this study.

BCMA CAR NK92 cells led tumor regression in an NCI-H929-derived MM subcutaneous tumor model without causing serious side effects. A subcutaneous tumor model was established with NCI-H929, a BCMA-positive MM cell line. Surely, BCMA CAR NK92 cells induced obvious tumor remission in a MM subcutaneous tumor model, indicating their potentially therapy (Figure 5). These results demonstrate the therapeutic efficacy of BCMA CAR NK92 cells in immunotherapy of MM. These findings indicate the contribution of BCMA CAR NK92 cells to antitumor immune responses in clinical trials.

Our current study has a few limitations. In vivo studies show reduced tumor regression only in the NCI-H929 subcutaneous model. Therefore, a metastatic model or another tumor model should be considered in the future. The NK92 cell line used in this study is easy to culture but requires irradiation before clinical use. Most current clinical trials with NK cells administer autologous or allogeneic ex vivo expanded NK cells. Thus, clinical trials of CAR NK immunotherapy using primary NK cells must be prepared.

In conclusion, BCMA-specific CAR NK92 cells are effective against MM cell lines and primary MM cells from patients. This study indicates that BCMA-specific CAR NK92 cells have great potential to kill MM cell by releasing cytokines, providing an experimental platform for new clinical therapies of BCMA-specific CAR-modified NK cells for treating MM. They could also be expanded to research autologous or allogeneic primary NK cells based on NK cell lines. 

## Data Availability

The data presented in this study are available on request from the corresponding author.

## References

[1] Feng D., Sun J. (2020). Overview of anti-BCMA CAR-T immunotherapy for multiple myeloma and relapsed/refractory multiple myeloma. Scand. J. Immunol..

[2] Garg A., Aggarwal M., Kashyap R. (2021). Pregnancy and Its Successful Outcome in a Patient with Multiple Myeloma. J. Obstet. Gynaecol. India.

[3] Lee J.H., Kim S.H. (2020). Treatment of relapsed and refractory multiple myeloma. Blood Res..

[4] Chen D., Frezza M., Schmitt S., Kanwar J., Dou Q.P. (2011). Bortezomib as the First Proteasome Inhibitor Anticancer Drug: Current Status and Future Perspectives. Curr. Cancer Drug Targets.

[5] Goldman-Mazur S., Visram A., Kapoor P., Dispenzieri A., Lacy M.Q. (2023). Outcomes after biochemical or clinical progression in patients with multiple myeloma. Blood Adv..

[6] Pan K., Farrukh H., Veera CSRChittepu Xu H., Pan C.X., Zhu Z. (2022). CAR race to cancer immunotherapy: From CAR T, CAR NK to CAR macrophage therapy. J. Exp. Clin. Cancer Res..

[7] Abbasi S., Totmaj M.A., Abbasi M., Hajazimian S., Goleij P. (2023). Chimeric antigen receptor T (CAR-T) cells: Novel cell therapy for hematological malignancies. Cancer Med..

[8] Sharma P., Kumar P., Sharma R. (2017). Natural Killer Cells—Their Role in Tumour Immunosurveillance. J. Clin. Diagn. Res..

[9] Smyth M.J., Hayakawa Y., Takeda K., Yagita H. (2002). New aspects of natural-killer-cell surveillance and therapy of cancer. Nat. Rev. Cancer.

[10] Hasegawa T., Suresh V.V., Yahata Y., Nakano M., Suzuki S. (2021). Inhibition of the CXCL9-CXCR3 axis suppresses the progression of experimental apical periodontitis by blocking macrophage migration and activation. Sci. Rep..

[11] Zhang Q., Xu J., Ding J., Liu H., Li H. (2018). Bortezomib improves adoptive carbonic anhydrase IX-specific chimeric antigen receptor-modified NK92 cell therapy in mouse models of human renal cell carcinoma. Oncol. Rep..

[12] Zhang C., Oberoi P., Oelsner S., Waldmann A., Lindner A., Tonn T., Wels W.S. (2017). Chimeric Antigen Receptor-Engineered NK-92 Cells: An Off-the-Shelf Cellular Therapeutic for Targeted Elimination of Cancer Cells and Induction of Protective Antitumor Immunity. Front. Immunol..

[13] Yu B., Jiang T., Liu D. (2020). BCMA-targeted immunotherapy for multiple myeloma. J. Hematol. Oncol..

[14] Dogan A., Siegel D., Tran N., Fu A., Fowler J., Belani R., Landgren O. (2020). B-cell maturation antigen expression across hematologic cancers: A systematic literature review. Blood Cancer J..

[15] Paul S., Lal G. (2017). The Molecular Mechanism of Natural Killer Cells Function and Its Importance in Cancer Immunotherapy. Front. Immunol..

[16] Molfetta R., Quatrini L., Santoni A., Paolini R. (2017). Regulation of NKG2D-Dependent NK Cell Functions: The Yin and the Yang of Receptor Endocytosis. Int. J. Mol. Sci..

[17] Long E.O., Kim H.S., Liu D., Peterson M.E., Rajagopalan S. (2013). Controlling natural killer cell responses: Integration of signals for activation and inhibition. Annu. Rev. Immunol..

[18] Fauriat C., Long E.O., Ljunggren H.G., Bryceson Y.T. (2010). Regulation of human NK-cell cytokine and chemokine production by target cell recognition. Blood.

[19] Kawano Y., Fujiwara S., Yuki H., Tatetsu H., Yamasaki H. (2011). Decreased CD138 Expression in Myeloma Cells: A Potential Indicator of Poor Prognosis and Aberrant Differentiation. Blood.

[20] Field-Smith A., Morgan G.J., Davies F.E. (2006). Bortezomib (Velcade™) in the Treatment of Multiple Myeloma. Ther. Clin. Risk Manag..

[21] Kleber M., Ntanasis-Stathopoulos I., Terpos E. (2021). BCMA in Multiple Myeloma—A Promising Key to Therapy. J. Clin. Med..

[22] Banerjee R., Lee S.S., Cowan A.J. (2022). Innovation in BCMA CAR-T therapy: Building beyond the Model T. Front. Oncol..

